# Early Clinical Outcomes of Full-Arch Rehabilitations with Immediately Loaded Implants with Buccal Dehiscence Treated with Horizontal Augmentation: A 1-Year Retrospective Case Series

**DOI:** 10.3390/dj14020121

**Published:** 2026-02-19

**Authors:** Alfonso Acerra, Mario Caggiano, Angelo Aliberti, Michele Langone, Francesco Giordano

**Affiliations:** 1 Department of Medicine, Surgery and Dentistry, University of Salerno, 84081 Salerno, Italy; 2 Department of Neurosciences, Reproductive Sciences and Odontostomatological Sciences, University of Naples Federico II, 80131 Naples, Italy

**Keywords:** buccal bone dehiscence, horizontal bone augmentation, immediate loading, full-arch rehabilitation, retrospective case series

## Abstract

**Background**: Buccal bone dehiscence is a frequent finding during implant placement and often requires horizontal bone augmentation. When combined with immediate loading protocols, concerns remain regarding early implant stability and failure risk. This retrospective case series aimed to describe the early clinical outcomes of immediately loaded implants placed in sites with buccal dehiscence treated by horizontal bone augmentation and restored with full-arch screw-retained prostheses. **Methods**: Fifty-nine consecutive edentulous patients were rehabilitated with immediately loaded cross-arch implant-supported prostheses. A total of 253 implants were placed, including 148 implants presenting buccal dehiscence and treated with horizontal bone augmentation using particulate grafting materials with or without autogenous bone and a resorbable collagen membrane. Clinical outcomes were assessed over a 1-year follow-up period. Implant survival and biological complications were recorded. Descriptive statistics were applied. An exploratory event-based comparison between augmented and non-augmented implants was performed using Fisher’s exact test, and risk ratios (RRs) with 95% confidence intervals (CIs) were calculated. **Results**: At 1 year, no patients were lost to follow-up. Two implant failures occurred, both in augmented sites (2/148; 1.35%), while no failures were observed among non-augmented implants (0/105). The exploratory comparison did not show a statistically significant difference in failure rates between groups (*p* = 0.51). The estimated RR for implant failure associated with horizontal augmentation was 3.56 (95% CI: 0.17–73.34). Two biological complications (one peri-implantitis and one peri-implant mucositis) were recorded, both involving augmented implants. **Conclusions**: Within the limitations of this retrospective case series, immediately loaded implants placed in sites with buccal dehiscence and treated with horizontal bone augmentation demonstrated high early survival rates and a low incidence of biological complications. These findings are descriptive and exploratory and should be interpreted as hypothesis-generating. Further prospective controlled studies with longer follow-up are needed to confirm these observations.

## 1. Introduction

Despite continuous advances in preventive and restorative dentistry, edentulism remains a prevalent clinical condition that significantly affects oral function, facial aesthetics, and patients’ quality of life [1,2]. Fixed implant-supported rehabilitations are widely accepted as a treatment modality for fully edentulous patients, offering predictable functional and esthetic outcomes [3]. In particular, full-arch screw-retained prostheses supported by four to six implants have demonstrated high survival rates and favorable long-term results in both the maxilla and mandible [4,5,6].

A common approach for full-arch rehabilitation involves implant placement within the interforaminal region of the mandible or the intersinus area of the maxilla [7,8]. However, anatomical constraints such as progressive alveolar bone resorption, superficial positioning of the mental foramen, and maxillary sinus pneumatization may limit optimal implant positioning [9,10]. To overcome these limitations, distal implants are often placed with intentional angulation in order to increase the anteroposterior spread, improve load distribution, and reduce prosthetic cantilever length [11,12]. While tilted implant configurations offer biomechanical advantages, they may also introduce additional prosthetic and biological challenges that require careful surgical and restorative planning [13,14]. A cross-sectional study by Gibello et al. evaluating full-arch implant-supported prostheses showed that the prosthetic complication rate increased over the follow-up period, despite a 100% implant survival rate. These findings suggested that prosthetic complication rates tend to increase with longer durations of prosthesis function [15].

A comprehensive preoperative assessment is therefore essential, particularly with regard to the evaluation of available hard tissue volume and the potential presence of buccal bone defects [16].

Buccal bone dehiscences and fenestrations are frequently encountered during implant placement and are defined as partial or complete absence of the buccal cortical plate, resulting in exposure of the implant surface [17]. These defects may arise from pre-existing alveolar ridge resorption, post-extraction remodeling, or individual anatomical variations [18,19]. When present, such defects represent a significant surgical challenge and may compromise implant stability, peri-implant tissue health, and long-term outcomes if left untreated [20]. Consequently, horizontal bone augmentation procedures are commonly indicated to restore the buccal bone contour and promote favorable conditions for osseointegration [21,22].

Horizontal guided bone regeneration typically involves the use of particulate grafting materials, either alone or in combination with autogenous bone, often covered by a resorbable collagen membrane [23]. This approach aims to re-establish adequate hard tissue support, protect the implant surface, and enhance both functional and esthetic outcomes. However, these regenerative procedures are technique-sensitive, and their application in conjunction with immediate loading protocols raises additional clinical concerns [24].

Immediate loading has gained increasing popularity due to reduced treatment time, improved patient comfort, and faster functional rehabilitation [25,26]. Nevertheless, its success is closely associated with achieving adequate primary implant stability, which is commonly assessed through insertion torque values or implant stability quotient measurements [27,28]. When implants are placed in sites requiring simultaneous bone augmentation, particularly in the presence of buccal dehiscence, the mechanical engagement with native bone may be reduced. This condition could potentially compromise primary stability and increase the risk of early implant failure or biological complications [29].

Despite the widespread clinical use of immediate loading protocols and horizontal augmentation techniques, limited data are available regarding their combined application in implants placed in sites with buccal dehiscence. Most available evidence derives from heterogeneous clinical reports, and the early outcomes of such combined approaches remain insufficiently documented.

Therefore, the aim of this retrospective case series was to describe the early clinical outcomes of immediately loaded implants placed in sites with buccal dehiscence treated by horizontal bone augmentation and restored with full-arch screw-retained prostheses. By reporting implant survival and early biological complications over a one-year follow-up period, this study seeks to provide descriptive, hypothesis-generating data to support clinical decision making and to inform future prospective investigations.

## 2. Materials and Methods

### 2.1. Study Design and Ethical Considerations

This investigation was designed as a retrospective observational case series. Consecutive edentulous patients rehabilitated with immediately loaded full-arch implant-supported prostheses, in whom at least one implant exhibited a buccal bone dehiscence at the time of placement, were included. Clinical data were collected from patients treated in a private dental practice in Sala Consilina (Salerno, Italy) by a single experienced clinician (F.G.) between January 2014 and November 2019.

All procedures were conducted in accordance with the principles of the Declaration of Helsinki. All patients provided written informed consent for treatment and for the use of their anonymized clinical data for scientific purposes. This study was reported following the STROBE (Strengthening the Reporting of Observational Studies in Epidemiology) guidelines for observational studies.

According to local regulations governing retrospective observational studies based on anonymized clinical records, formal ethics committee approval was not required.

### 2.2. Patient Selection Criteria

Patients were eligible if they met the following inclusion criteria, summarized in Table 1.

Four to six implants with a minimum length of 11 mm and a minimum diameter of 4.5 mm were placed in the presence of adequate bone volume. Immediate implants were included. Exclusion criteria were as follows in Table 2.

Patients were classified as non-smokers or smokers (≤10 cigarettes/day) and according to opposing dentition (natural/fixed prostheses or complete dentures).

### 2.3. Pre-Operative Planning and Surgical Protocol

Initial diagnostic assessment was performed using panoramic radiographs, employing radiopaque markers as reference points to assist in anatomical orientation. To obtain the requisite detail for definitive treatment planning, Cone-Beam Computed Tomography (CBCT) scans (Carestream Dental LLC, Atlanta, GA, USA) were acquired with the radiographic guide in situ, aiding in both virtual planning and surgical navigation. These same guides were subsequently employed intraoperatively to guarantee precise implant placement, thereby strictly adhering to the established preoperative digital planning. Prior to surgery, all patients underwent professional oral prophylaxis and received 2 g of amoxicillin combined with clavulanic acid administered orally one hour prior to the procedure. Penicillin-allergic individuals were prescribed a single dose of 500 mg clarithromycin one hour before the procedure. Immediately before surgery, patients rinsed for one minute with a 0.2% chlorhexidine gluconate solution. Conscious sedation was administered intravenously using a combination of sedatives, followed by local anesthesia using mepivacaine with epinephrine at concentrations of 1:100,000 or 1:50,000. The design of the mucoperiosteal flap was executed utilizing full-thickness crestal incisions. In cases of adequate hard tissue volume, limited mucoperiosteal elevation was performed. Conversely, when bone availability was compromised, extended full-thickness flaps were raised to expose critical anatomical structures, such as the mental foramen or the anterior maxillary sinus wall. Any residual teeth or root fragments were removed using atraumatic techniques, and surgical sites were thoroughly degranulated.

Implant placement involved the use of conical-cylindrical SPI-Contact implants (Thommen Medical; Grenchen, Switzerland), featuring a 1.5 mm polished collar. Implants were predominantly 11 mm in length and 4.5 mm in diameter. All implants were placed according to the manufacturer’s guidelines, except for site preparation: a 4.0 mm profile drill was used in place of the recommended size to intentionally underprepare the osteotomy and thereby enhance primary stability through increased insertion torque. In the premolar regions, distal implants were positioned with a distal angulation to optimize load distribution.

Buccal dehiscence was defined intraoperatively as a partial or complete absence of the buccal cortical plate resulting in exposure of the implant surface after implant placement and flap elevation. Due to the retrospective nature of this study, defect morphology (e.g., standardized measurements of defect width and depth) was not systematically recorded in a reproducible manner and therefore could not be included in the analyses.

Implants were placed with the polished collar supracrestally positioned, even in post-extraction scenarios. A motor-driven insertion torque of 70 Ncm was applied, after which primary stability was verified manually using the SPI-MONO torque ratchet (Thommen Medical; Grenchen, Switzerland). If any of the initially planned four implants failed to reach a minimum insertion torque of 35 Ncm, an additional implant (fifth or sixth) was placed adjacent to the low-stability unit. The 35 Ncm threshold was adopted as a pragmatic criterion commonly used to support immediate loading protocols, as suggested in the literature [30]. In cases where two or more implants failed to achieve sufficient torque, the surgical protocol dictated submerging all implants and deferring prosthetic loading for a three-month osseointegration period [25].

**Figure 1:**
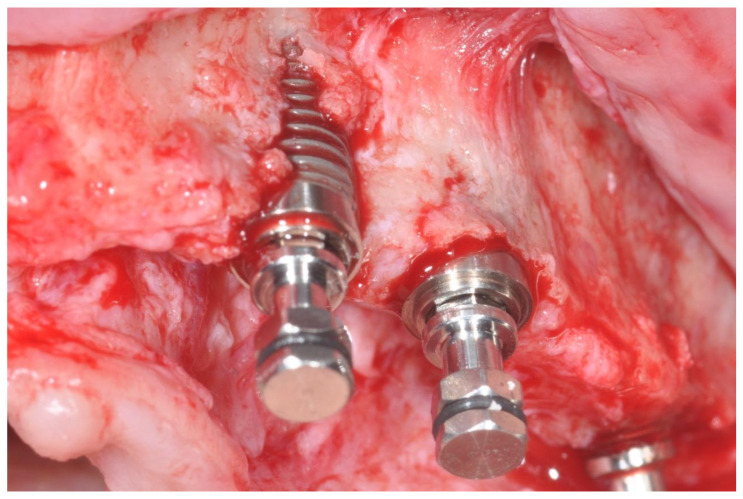
Bone dehiscence of the buccal alveolar bone.

In cases of buccal bone dehiscence (Figure 1), an autogenous granular bone layer was harvested using a bone scraper (14620.10 Stoma; Emmingen-Liptingen, Germany) and applied to cover the exposed implant surface. This graft was subsequently overlaid with an inorganic bovine bone substitute (Bio-Oss, Geistlich, Wolhusen, Switzerland). A double layer of resorbable collagen membrane (Bio-Gide, Geistlich, Switzerland) was then placed over the graft and stabilized with titanium pins, initially using Kalos pins (Orbetello, Italy) and later Smartact pins (Meta, Reggio Emilia, Italy). This regenerative protocol was implemented in 23 implants, whereas in 125 implants only the inorganic bovine bone graft was utilized without the autogenous one. Following graft placement, healing abutments were installed, and wound closure was achieved using single resorbable sutures (Vicryl 4-0 SH1, 22 mm 1/2 c; Ethicon, New Brunswick, NJ, USA).

### 2.4. Post-Operative Management and Prosthetic Protocol

Postoperatively, patients were advised to take analgesics (Ibuprofen 600 mg) twice daily with meals. A soft diet was recommended for 45 days. Professional oral hygiene inspection was conducted over the three days following surgery to facilitate prosthesis preparation. Patients were instructed to refrain from brushing the surgical area and from rinsing until suture removal, which occurred approximately 10 days after the procedure. Cleaning of the prostheses was recommended using gauze soaked with 0.2% chlorhexidine solution, followed by progressive use of a soft toothbrush, then a medium-bristled toothbrush, and ultimately a water jet device. The definitive prosthetic rehabilitation was initiated immediately following implant insertion.

Individual perforated trays were used to take impressions with precision material. Impregum F (Espe Dental AG, Seefeld, Germany). Definitive screw-retained prostheses were made by placing titanium abutments (VARIOtemp for fixed prostheses, Thommen) on the model, which were connected using titanium rods of 2 mm diameter soldered with an argon syncrystallization device (WELDER INTRAORAL MIDI, Implamed, Cremona, Italy), an intraoral welder, to create a rigid framework. Despite the disparallelism between the implants, it was not necessary to use angulated abutments on the tilted implants. On the second post-operative day, the framework was intraorally evaluated to meticulously assess its adaptation, verify the aesthetic and functional parameters, and confirm phonetic integration.

It was then finalized with a lining in acrylic resin, and the inter-arch relationship was recorded based on the pre-operative digitally designed surgical-prosthetic template. A cantilever length of no more than 1.5 cm per side was permitted. On the third day after surgery, prostheses were screwed onto the implants using a standard torque of 25 Ncm, and the screw access holes were sealed with gutta-percha. A panoramic radiograph was obtained to check the precise seating and secure adaptation of the definitive abutments onto the implants.

Prostheses were designed to have a group function occlusal scheme and were adjusted to have homogeneous occlusal contacts also on cantilevers, when present. Suture removal was performed approximately ten days post-operatively by cutting the knot and leaving the remaining portion of the absorbable suture inside the soft tissues to be spontaneously resorbed. Detailed post-operative oral hygiene protocols were comprehensively communicated to the patient.

### 2.5. Follow-Up

**Figure 2:**
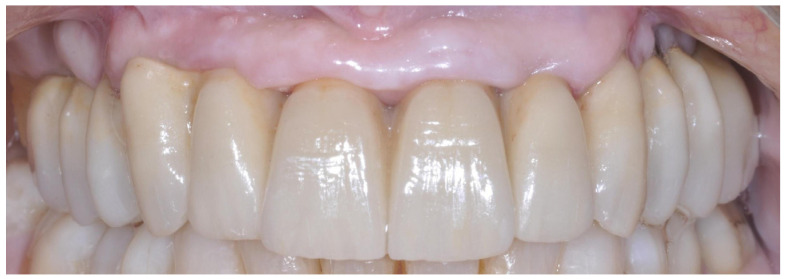
Definitive screw-retained prosthesis.

Patients were seen again every month for the first six months. After a three-month follow-up period, the prostheses were rebased. Patients were recalled for maintenance visits (Figure 2), and the occlusion was evaluated every six months, during which the screw-retained prostheses were removed to assess implant stability and allow for professional cleaning. Patient compliance with home oral hygiene guided the follow-up period.

### 2.6. Outcome Evaluation

The outcomes evaluated in this study were as follows:

- Implant failure: Any mobility and/or infection requiring implant removal and/or biomechanical complications such as implant fracture or deformations of the connections. Implant stability was checked at baseline, 1 month, 3 months after implant placement and thereafter every 6 months by removing the screw-retained prosthesis and checking implant mobility by rocking test through handling of two instruments;
- Any complications, including bleeding, numbness of the lower lip and chin, peri-implant mucositis or peri-implantitis, fistulas formation, screw loosening, phonetic problems, etc.

This study was structured as a retrospective single-cohort study. The inclusion criteria were defined to encompass patients exhibiting alveolar bone dehiscence, who subsequently underwent horizontal guided bone regeneration and rehabilitation with a fixed, screw-retained cross-arch prosthesis planned for immediate functional loading.

No sample size calculation was performed. Patients were considered the statistical unit for descriptive analyses, whereas implant-level analyses were adopted for event-based outcomes.

### 2.7. Statistical Analysis

Due to the retrospective nature of the study design, neither an a priori sample size determination nor a statistical power analysis was performed. Inferential statistical testing was not planned a priori, given the absence of a formal control group and the primary descriptive aim of this study. Therefore, results should be interpreted as exploratory and hypothesis-generating rather than confirmatory.

Nevertheless, an exploratory post hoc comparison was subsequently performed, as described below.

Descriptive statistics were used to summarize patient- and implant-level characteristics, including frequencies and percentages for categorical variables. Patient-level variables were used to describe the study population, whereas implant-level analyses were adopted for event-based outcomes, including implant failure and biological complications.

In order to explore potential differences in early implant failure occurrence, an event-based comparison was performed between augmented and non-augmented implants. Given the low number of events and the presence of zero-event cells, Fisher’s exact test was selected as the most appropriate method for comparing failure rates between groups.

To quantify the magnitude of association between horizontal augmentation and implant failure, risk ratios (RRs) with 95% confidence intervals (CIs) were calculated. A continuity correction (Haldane–Anscombe method) was applied to allow estimation in the presence of zero failures in one group.

All statistical analyses were conducted at the implant level and were considered exploratory in nature. No adjustment for multiple comparisons was performed, and statistical significance was not the primary objective of the analysis. Because multiple implants were placed in the same patient, implant-level outcomes may not be statistically independent; no adjustment for within-patient clustering (e.g., mixed-effects models or robust standard errors) was performed due to the very low number of events. Therefore, implant-level comparative results should be interpreted as hypothesis-generating. A two-sided *p*-value < 0.05 was considered indicative of statistical significance. Analyses were performed using standard statistical software.

## 3. Results

### 3.1. Patient- and Implant-Level Characteristics

A total of 59 consecutive edentulous patients (32 females and 27 males) with a mean age of 58 years (range: 40–80 years) were included. No patients were lost to follow-up during the 1-year observation period.

Overall, 59 immediately loaded full-arch screw-retained prostheses (45 maxillary and 14 mandibular) were delivered on 253 implants. Among these, 148 implants (58.5%) presented a buccal bone dehiscence at the time of placement and were treated with horizontal bone augmentation, while 105 implants (41.5%) were placed in sites without dehiscence and did not require augmentation. Baseline patient characteristics and treatment-related variables are summarized in Table 3.

Among the augmented implants, 112 were located in the maxilla and 36 in the mandible. A total of 114 implants were placed in post-extractive sites, of which 77 exhibited buccal dehiscence. Overall, 42 implants with dehiscence were placed with an insertion torque ≤ 35 Ncm.

### 3.2. Implant Survival and Clinical Outcomes

After 1 year of functional loading, two implant failures were recorded, resulting in an overall implant survival rate of 99.2%. Both failures occurred in augmented sites, corresponding to a failure rate of 1.35% among augmented implants (2/148). No implant failures were observed among non-augmented implants (0/105).

Both failed implants were located in the maxilla and occurred in two different patients. One failure involved an implant placed with an insertion torque ≤ 35 Ncm, whereas the second failure occurred in an implant placed with an insertion torque > 35 Ncm. A detailed description of the failed implants, including patient-related characteristics, implant position, insertion torque, and timing of failure, is provided in Table 4. No mechanical complications, such as implant fracture or prosthetic failure, were recorded during the follow-up period.

### 3.3. Biological Complications

Two biological complications were observed during the 1-year follow-up period, both affecting augmented implants. Specifically, one case of peri-implantitis and one case of peri-implant mucositis were recorded, corresponding to a complication rate of 1.35% among augmented implants. No biological complications were observed in non-augmented sites.

Details regarding the type of complication and implant location are reported in Table 5.

### 3.4. Results of Statistical Analysis

An exploratory event-based comparison of implant failure rates between augmented and non-augmented implants was performed. Given the low number of failures and the presence of zero-event cells, Fisher’s exact test was used.

The analysis did not demonstrate a statistically significant difference in early implant failure rates between augmented and non-augmented implants (*p* = 0.51). The estimated risk ratio for implant failure associated with horizontal bone augmentation was 3.56, with a wide 95% confidence interval (0.17–73.34), reflecting the limited number of events and reduced statistical precision. In addition, exact binomial 95% confidence intervals were calculated for overall implant survival and group-specific failure proportions to provide an estimate of uncertainty around descriptive rates.

No additional implant- or patient-level variables were formally tested for association with implant failure due to the small number of observed events.

## 4. Discussion

This retrospective case series evaluated the 1-year clinical outcomes of immediately loaded implants placed in sites presenting buccal bone dehiscence and treated with horizontal bone augmentation. The primary finding of the present study was a high overall implant survival rate (99.2%), with only two implant failures and two biological complications observed during the follow-up period. These outcomes are consistent with previously published reports on immediate loading protocols in full-arch rehabilitations [25,31,32].

The management of buccal bone dehiscence represents a frequent and clinically relevant challenge in implant dentistry, particularly in post-extractive sites and in the anterior maxilla, where buccal plate resorption is common [33,34,35]. Immediate implant placement in such conditions has been associated with an increased risk of soft tissue recession and esthetic complications if dehiscence defects are not adequately managed [36,37,38]. Horizontal bone augmentation using particulate grafts and resorbable collagen membranes is widely considered a predictable technique to restore buccal bone volume and to support peri-implant tissues [39,40,41]. Long-term retrospective and longitudinal studies have demonstrated favorable outcomes for horizontal ridge augmentation procedures, including satisfactory bone regeneration and stability over time [42,43,44].

From a contemporary implantology perspective, insertion torque provides only an indirect estimate of primary stability at the time of implant placement. Resonance Frequency Analysis (RFA), expressed as Implant Stability Quotient (ISQ), represents a non-invasive method to objectively monitor implant stability over time and to support clinical decision making regarding loading protocols [45]. ISQ values range from 0 to 100, and values approximately between 60 and 70 are often considered clinically meaningful for assessing primary and secondary stability in many clinical settings. Although RFA was not available in the present retrospective series, future prospective studies combining insertion torque measurements with serial ISQ assessments could provide a more comprehensive evaluation of stability dynamics in augmented dehiscence sites undergoing immediate loading.

Despite the favorable overall outcomes observed in the present study, it is noteworthy that all implant failures occurred in sites treated with horizontal augmentation. One failed implant exhibited low insertion torque (<35 Ncm), reinforcing the importance of primary stability in immediate loading protocols [46]. Nevertheless, the occurrence of a failure in an implant placed with adequate insertion torque suggests that additional factors may influence early implant failure in augmented sites. The biological and biomechanical characteristics of regenerated bone, which often consists of a combination of autogenous and xenogeneic graft materials, may differ from those of pristine bone and potentially affect early osseointegration [47,48].

Defect morphology and graft composition may represent additional factors influencing early outcomes in augmented dehiscence sites. Wider or deeper buccal defects may reduce native bone–implant contact, potentially affecting primary stability and biomechanical behavior during immediate loading. Moreover, in the present series, some implants were treated using a thin autogenous bone layer combined with an anorganic bovine bone substitute, whereas others received the xenograft alone. Such differences in graft composition may have influenced early healing dynamics and the biological environment surrounding the implants. However, the retrospective design, the absence of standardized defect measurements, and the low number of adverse events did not allow meaningful subgroup or comparative analyses.

The exploratory statistical analysis performed did not reveal a statistically significant difference in early implant failure rates between augmented and non-augmented implants. However, the wide confidence interval associated with the estimated risk ratio reflects the limited number of events and the consequent lack of statistical precision. Therefore, the absence of statistical significance should not be interpreted as evidence of equivalence between the two groups but rather as a limitation inherent to the exploratory nature of the analysis.

Biological complications were also limited to augmented implants, with one case of peri-implantitis and one case of peri-implant mucositis recorded during the follow-up period. These findings may be related to the increased complexity of maintaining peri-implant tissue health in regenerated sites, where altered soft tissue contours and grafted bone may complicate plaque control [49,50]. In this context, increasing attention has been directed toward biological adjuvants capable of enhancing soft tissue healing and early wound stabilization in oral surgery [51]. Autologous platelet concentrates have been extensively investigated for their potential to improve clinical and radiographic healing following tooth extractions and impacted third molar removal, demonstrating favorable effects on postoperative recovery and soft tissue outcomes [52,53]. Although these approaches were not applied in the present study, their biological rationale may be of interest for future investigations aiming to optimize peri-implant tissue healing, particularly in surgically complex scenarios involving bone augmentation.

Previous studies have highlighted the importance of supportive care and patient compliance in preventing peri-implant diseases, particularly in complex rehabilitations involving immediate loading and bone augmentation procedures [54,55].

Immediate implant placement and loading in the presence of buccal bone dehiscence can be considered a viable treatment option in selected cases; however, wider or deeper defects have been associated with an increased risk of gingival recession and biological complications [56,57]. This underscores the importance of careful case selection, accurate intraoperative assessment of defect morphology, and the use of appropriate hard- and soft-tissue augmentation techniques.

The clinical decision-making process during surgery plays a crucial role in the management of dehiscence defects. Early identification of buccal bone defects requires surgical experience and the ability to adapt the treatment plan accordingly. Proceeding with simultaneous horizontal augmentation and immediate loading should be based on a comprehensive evaluation of implant primary stability, bone quality, defect extension, and patient-related risk factors such as smoking or systemic conditions.

Compared with other clinical reports, the present study benefits from a relatively large number of implants placed according to a standardized surgical and prosthetic protocol and treated by a single experienced clinician. In addition, no patients were lost to follow-up, strengthening the internal consistency of the data.

### Limitations of This Study

Several limitations of the present study must be acknowledged. First, the retrospective observational design inherently limits the ability to control for confounding variables and precludes causal inference. No formal control group was included, and the analysis was primarily descriptive in nature. Although an exploratory statistical comparison was performed, the very low number of implant failures limited the possibility of identifying statistically meaningful associations or predictive factors. Additionally, implant-level analyses may be affected by within-patient clustering because multiple implants were placed in the same individual; no clustering adjustment was applied, which may limit inferential interpretation of implant-level comparisons.

Second, no a priori sample size calculation or statistical power analysis was performed, and the patient was considered the statistical unit for descriptive purposes. This limits the generalizability of the findings and the interpretation of subgroup analyses.

Third, implant stability was assessed clinically using a manual rocking test rather than quantitative methods such as resonance frequency analysis. While this assessment was consistently performed by the same experienced clinician, it may introduce a degree of subjectivity.

In addition, standardized radiographic evaluation of marginal bone levels was not available, preventing quantitative assessment of peri-implant bone changes over time. This was due to the retrospective nature of this study and the lack of standardized longitudinal radiographic imaging protocols.

Finally, the 1-year follow-up period allows evaluation of early implant survival and short-term biological outcomes but does not permit conclusions regarding long-term implant performance, peri-implant bone stability, or prosthetic maintenance.

## 5. Conclusions

Within the limitations of this retrospective case series, implants placed in sites presenting buccal bone dehiscence and treated with horizontal bone augmentation demonstrated high early survival rates and a low incidence of biological complications when immediately loaded under full-arch screw-retained prostheses.

Although all implant failures and biological complications occurred in augmented sites, the overall frequency of adverse events was low during the 1-year follow-up period. These findings suggest that, under stringent clinical conditions and careful case selection, the combination of horizontal augmentation and immediate loading may represent a feasible therapeutic approach in selected edentulous patients.

Nevertheless, given the retrospective design, the short follow-up period, and the exploratory nature of the statistical analysis, the results should be interpreted with caution. Further prospective controlled studies with larger sample sizes, objective outcome measures, and longer follow-up are required to confirm the clinical predictability and long-term performance of this treatment protocol.

## Figures and Tables

**Table 1:** Inclusion criteria.

Inclusion Criteria
Complete edentulism in one or both arches
Availability of bone to place at least four implants, each ≥11 mm in length and ≥4.5 mm in diameter
Presence of at least one implant site exhibiting a buccal dehiscence requiring horizontal augmentation
Possibility of delivering an immediate screw-retained metal-resin cross-arch prosthesis

**Table 2:** Exclusion criteria.

Exclusion Criteria
General contraindications to implant surgery
History of cardiovascular events within the last 6 months
Patients with a compromised or suppressed immune system
Patients with a blood glucose level > 150 mg/dL
pregnant or breastfeeding patients
Irradiated in the head/neck in the previous 6 months
Treated or under treatment with intravenous amino-bisphosphonates
Inadequate oral hygiene or lack of patient compliance
Active infection or severe inflammation in the area intended for implant placement
Absence of opposing teeth/prosthetic restorations

**Table 3:** Patients’ and intervention characteristics.

Patients’	Numbers
Females	32 (54.2%)
Males	27 (45.8%)
Mean age at implant insertion (range)	58 (40–80)
Non-smokers	43 (73%)
Smoking up to 10 cigarettes/day	16 (27%)
Patients with a positive general anamnesis	1 (1.7%)
Patients wearing dentures in the opposite jaw at implant loading	2 (3.7%)
**Intervention characteristics**	
Total number of inserted implants	253
Implants inserted in mandibles (14 jaws)	57 (22.5%)
Implants inserted in maxillae (45 jaws)	196 (77.5%)
Implants with dehiscence in mandibles	36 (24%)
Implants with dehiscence in maxillae	112 (76%)
Implants in extraction sockets	114 (45%)
Implants in extraction sockets with dehiscence	77 (30.5%)
Implants in extraction sockets without dehiscence	37 (14.6%)
Implants placed with a torque ≤ 35 Ncm in dehiscence only	42 (16.6%)

**Table 4:** Description of the implant failures occurred up to the first year of loading.

Patients	Patient’s Characteristics	Implant Position Characteristics and Failure Timing	Symptoms
2	55 y.o. male, non-smoker	2.2 augmented dehiscence, torque < 35 Ncm, 9 *	none
33	74 y.o. male, smoker	1.5 augmented dehiscence, torque > 35 Ncm, 10 *	none

* months post-loading.

**Table 5:** Description of the complications that occurred up to the first year of function.

Number of Patients	Type of Complication	Implant Position Characteristics
2	peri-implantitis	1.2 augmented dehiscence
6	peri-implant mucositis due to plaque accumulation	3.6 augmented dehiscence

## Data Availability

The original contributions presented in this study are included in the article. Further inquiries can be directed to the corresponding authors.
